# Evaluation of Antioxidant and Antibacterial Effects of Lyophilized Cell-Free Probiotic Supernatants of Three *Lactobacillus* spp. and Their Cytocompatibility Against Periodontal Ligament Stem Cells

**DOI:** 10.5812/ijpr-136438

**Published:** 2023-07-11

**Authors:** Maryam Torshabi, Mohammad Mahdi Bardouni, Atieh Hashemi

**Affiliations:** 1 Department of Dental Biomaterials, School of Dentistry, Shahid Beheshti University of Medical Sciences, Tehran, Iran; 2 Department of Pharmaceutical Biotechnology, School of Pharmacy, Shahid Beheshti University of Medical Sciences, Tehran, Iran

**Keywords:** Periodontitis, Probiotics, Periodontal Ligament, Antioxidant Activity, Cytotoxicity

## Abstract

**Background:**

Periodontitis is a chronic disease characterized by the inflammation of the periodontium and leads to progressive damage, such as gingival atrophy, alveolar bone loss, and tooth loss. *Streptococcus mutans* and *Aggregatibacter actinomycetemcomitans* are bacteria that support the occurrence of periodontitis via the ability to form biofilms or damage the alveolar bone and periodontal ligaments. On the other hand, periodontal ligament stem cells (PDLSCs) are cells with differentiation capability into osteoblasts or osteoblasts. Due to their role in periodontal homeostasis and regeneration, PDLSCs are considered to control periodontitis progression. However, probiotics are helpful microorganisms known to have antimicrobial and immune-regulating effects.

**Objectives:**

This study aimed to evaluate the antioxidant activity and antimicrobial effects of lyophilized cell-free supernatants (LCFSs) derived from three probiotic strains of *Lactobacillus* on *S. mutans* and *A. actinomycetemcomitans*. Moreover, the effect of these lyophilized supernatants was investigated on the viability and migration capability of PDLSCs.

**Methods:**

The antibacterial effects of LCFSs of three probiotic bacteria were investigated by determining the minimum inhibitory concentration and minimum bactericidal concentration. Then, the effect of LCFSs on the survival and migration of PDLSCs was investigated by the MTT method (at 24 and 72 hours) and scratch test (at 0, 24, and 48 hours), respectively. Finally, the antioxidant effect of LCFSs was assessed by the 2,2-diphenyl-1-picrylhydrazyl (DPPH) assay and ferric reducing/antioxidant power methods.

**Results:**

The antibacterial properties of different concentrations of acidic and neutral LCFSs derived from three studied probiotic bacteria on *S. mutans* and *A. actinomycetemcomitans* were observed within the range of 12.5 - 50% (v/v) (1/8 - 1/2 dilutions with culture medium). Although there were no significant toxic (~ 100% viability) and wound healing effects on PDLSCs when the cells were exposed to either acidic or neutral studied LCFSs in a concentration of 5% (v/v), they showed significant antioxidant activity (~ 90% DPPH inhibition and 0.5 mM Fe^2+^/L).

**Conclusions:**

The results revealed that 5% (v/v) 48-hour acidic and neutral supernatants of three studied probiotics might play a beneficial role in controlling periodontitis.

## 1. Background

Periodontitis is a chronic infectious disorder that jeopardizes the structural integrity of the soft tissues and bones supporting the teeth. The progression of periodontitis can be described as a vicious circle starting with the inflammation of the tissue’s surface that advances to deeper tissues as the bacteria penetrate the compromised barrier, amplifying the existing inflammation. The oral and dental tissue contains a variety of stem cell types, such as periodontal ligament stem cells (PDLSCs), which can develop into osteoblast cells and play a crucial role in bone regeneration (1). Maintaining the immunological homeostasis of PDLSCs is also critical because they release inflammatory mediators, including interleukins 1, 6, and 8, when exposed to pathogens, which are crucial for the emergence of infectious-inflammatory dental disorders, such as periodontitis (2).

Since periodontitis is a chronic inflammatory disease brought on by bacterial biofilm, treating it with antibiotics and scaling and root planing techniques have been utilized in the past to manage microbial biofilm and remove dental plaque. The evidence suggests that the homeostasis between microbes and the host’s cellular responses, especially the immune response, have a vital role in the prevention and control of periodontitis. However, probiotics have been viewed in recent years as a promising alternative to periodontal treatments due to their direct effect on bacteria and their immunomodulatory effects (3-6).

Probiotics are living and helpful microorganisms that play an important protective role in human health by the prevention of the adhesion, penetration, and invasion of pathogens into body cells, the secretion of antimicrobial substances, and the modulation of immune parameters. Direct and indirect effects are the two broad categories used to describe how probiotics function in the mouth. Direct effects include suppressing the synthesis of antibacterial compounds and dental plaque and biofilm. Nevertheless, the immune modulation, regulation of mucus permeability, and regulation of the microbial population to reduce oral pathogens are considered indirect effects (7). Studies have also revealed that the cell-free supernatant (CFS) of *Lactobacillus* probiotics has effects on oral (8, 9) and non-oral cells that are anti-inflammatory, antioxidant, and cell-protective (10).

Most of the common microbial flora in the mouth and intestines include bacteria that produce lactic acid. These microbes can actively boost the immune system and preserve bodily health. For research purposes, probiotics can be utilized in the form of live bacteria, heat-killed bacteria, or CFSs. However, probiotic supplementation in the form of live bacteria might cause an infection, despite being a part of the body’s normal flora. Therefore, the lysate and CFS of probiotics are recommended to mitigate the risk of infection and even increase the shelf life of probiotics products (11).

## 2. Objectives

This experimental study aimed to compare the antioxidant activity and antimicrobial effects of the lyophilized cell-free supernatants (LCFSs) derived from three probiotics, including *Lactobacillus acidophilus*, *Lactobacillus casei*, and *Lactobacillus paracasei*, on the oral bacteria *Aggregatibacter actinomycetemcomitans* and *Streptococcus mutans*. Additionally, this study investigated the effect of LCFSs of three studied probiotic bacteria on the viability and migration of PDLSCs.

## 3. Methods

### 3.1. Bacterial Growth Curve

The studied bacteria (i.e., *L. acidophilus*, *L. casei*, and *L. paracasei*) were obtained from the Iranian Industrial Bacteria and Fungi Collection Center. To draw the growth curve of probiotic bacteria, the half-McFarland suspension was prepared from their 24-hour culture in a De Man, Rogosa, and Sharpe (MRS) broth medium (12). From the prepared suspension, 100 μL was inoculated into 50 mL of the MRS broth medium. The inoculated medium was kept in an anaerobic jar in an incubator (37°C), and 3, 6, 24, 30, 48, 54, and 72 hours later, sampling was performed to read the optical density (OD) at 600 nm and to culture on MRS agar medium for colony counting. Meanwhile, the pH of the environment was also measured during the sampling times (12).

### 3.2. Preparation of Cell-Free Supernatants

To isolate the CFSs of probiotic bacteria, a 1 McFarland suspension was prepared from their 24-hour culture in an MRS broth medium (12). Then, 100 μL of the prepared suspension was inoculated into 50 mL of the MRS broth medium. The inoculated medium was kept in an anaerobic jar in an incubator (37°C), and 20 mL of the supernatant was centrifuged (3000 g, 20 minutes) at 24 and 48 hours after cultivation. After filtration (0.22 µm), the pH of the supernatants was adjusted. Some supernatants were kept at the original acidic pH, and some were neutralized using sodium hydroxide (pH = 7.2). To concentrate and increase the stability and effectiveness of 24- and 48-hour acidic and neutral CFSs, 5 mL of each were freeze-dried. The LCFS was reconstituted in sterile distilled water (2 mL) and stored in a freezer at -20°C (12).

### 3.3. Evaluation of the Antibacterial Effect of LCFSs

The antibacterial effects of 24- and 48-hour acidic and neutral LCFSs derived from all three *Lactobacillus* probiotic bacteria on two studied oral bacteria, including *S. mutans* and periodontal bacteria *A. actinomycetemcomitans* were measured as minimum inhibitory concentration (MIC) and minimum bactericidal concentration (MBC) in brain heart infusion (BHI) broth/agar medium. For MIC determination, the broth microdilution method and resazurin colorimetry in a 96-well plate were used (12). The plates (treated with different dilutions of studied materials) were incubated at 37°C (i.e., anaerobic condition). After 24 hours of incubation, 10 µL resazurin dye solution (0.01%; Sigma-Aldrich, Germany) was added into all the wells; after 1 hour of incubation at 37°C, changing the blue color of resazurin to pink confirms the enzymatic activity of the growing bacteria. Therefore, the lowest concentration in which the color change was not observed (it remained blue) was considered MIC. To determine MBC, the samples were collected from all the wells with an antibacterial effect (indicating the lack of bacterial growth) using a sterile loop followed by culturing on BHI agar medium (three replicates for each sample). The above-mentioned plates were incubated at 37°C under anaerobic conditions (48 and 72 hours for *S. mutans* and *A. actinomycetemcomitans*, respectively). The lowest concentration of LCFS at which no colonies formed was considered MBC (12).

### 3.4. Evaluation of the Cytotoxic Effect of LCFSs

To investigate and compare the effect of 24- and 48-hour acidic and neutral LCFSs derived from three *Lactobacillus* probiotic bacteria (in concentrations from 0% (control) to 50% (v/v)) on the survival and proliferation of PDLSCs, the MTT colorimetric test was used (13). On the first day of the study, a 96-well plate (SPL Life Sciences, Korea) was seeded with 3,500 cells/well in Dulbecco’s Modified Eagle Medium (DMEM; Gibco, UK) and incubated in a humidified incubator for 24 hours at a temperature of 37°C and 5% CO_2_. On the second day of the study, cell treatment was performed with different concentrations of LCFS. The control group was the complete cell culture medium alone. Two series of identical plates were treated to check the acute (24 hours) and chronic (72 hours) cytotoxicity of LCFSs on PDLSCs. To perform the MTT assay, the plates corresponding to the designated testing time were removed from the incubator. After washing with phosphate-buffered saline buffer, a cell culture medium containing 10% MTT dye (Sigma-Aldrich, Germany) was introduced in each well. After 2 hours of incubation in a humidified incubator, the MTT dye was replaced with dimethyl sulfoxide (Sigma-Aldrich, Germany). The optical absorption of the resulting color was read at 570 and 620 nm using the plate reader (Anthus 2020, Austria).

### 3.5. Evaluation of the Wound Healing Effect of LCFSs

The scratch test was used to analyze the migration ability. In this case, on the first day of the study, a 24-well plate (SPL Life Sciences, Korea) was seeded with 10^5^ PDLSCs/well in DMEM and incubated in a humidified incubator for 24 hours at a temperature of 37°C and 5% CO_2_. On the second day, when the cells reached 100% confluence, a quick vertical scratch was made in each well with the help of a sterile 100-pipette tip (time 0). The cells were then treated with 48-hour LCFSs prepared at neutral pH. Then, 0 (moment of scratching), 24, and 48 hours of the scratch were monitored. The cells were stained with 0.1% crystal violet dye solution (Merck, Germany), photographed using an inverted microscope, and analyzed using Image J software (National Institutes of Health, Bethesda, MD, USA; version 1.5.3) (14).

### 3.6. Evaluation of the Antioxidant Effect of LCFSs by 2,2-Diphenyl-1-Picrylhydrazyl and Ferric Reducing/Antioxidant Power Assays

To investigate the antioxidant activity of LCFSs (24- and 48-hour LCFSs prepared in both acidic and neutral pH) with a concentration of 5% (v/v) (obtained from the MTT test), the 2,2-diphenyl-1-picrylhydrazyl (DPPH) antioxidant test was used (13). First, a 5% (v/v) concentration of LCFS, a 1 mM concentration of a vitamin C solution (positive control), and a 0.1 mM concentration of DPPH (Sigma-Aldrich, Germany) were prepared in absolute ethanol. For the test group preparation, 50 μL of each LCFS and vitamin C were added to 50 μL of DPPH dye solution in a 96-well culture plate (three replicates for each concentration). For the blank group preparation, 50 μL of any LCFS and vitamin C were added to 50 μL of absolute ethanol. The negative control reaction was prepared using absolute ethanol (50 μL) added to the DPPH dye solution (50 μL). After the incubation of the mixtures for 30 minutes at room temperature in the dark, the absorbance of the resulting solutions was quantified at 492 nm with a plate reader (Anthus 2020, Austria). The DPPH free radicals-scavenging activity was measured using the following formula:

100 × (ODc - (ODs - ODb))/ODc = DPPH free radical inhibition percentage

Where ODc is the absorption rate of the negative control, ODs is the test samples absorption rate, and ODb is the blank absorption rate.

In addition, to check the total antioxidant capacity of lyophilized supernatants, the Naxifer TM kit (ferric reducing/antioxidant power (FRAP) assay) was used following the manufacturer’s protocol (Navand Salamat, Iran).

### 3.7. Data Analysis

All the data were presented as mean ± standard deviation of at least three independent repeats. The statistical analyses of the obtained results were performed using GraphPad Prism software (version 9; La Jolla, CA, USA) by one-way analysis of variance and Tukey’s post-hoc test. The level of significance of the difference was considered < 0.05.

## 4. Results

### 4.1. Growth Curve of Probiotic Bacteria

**Figure 1.**
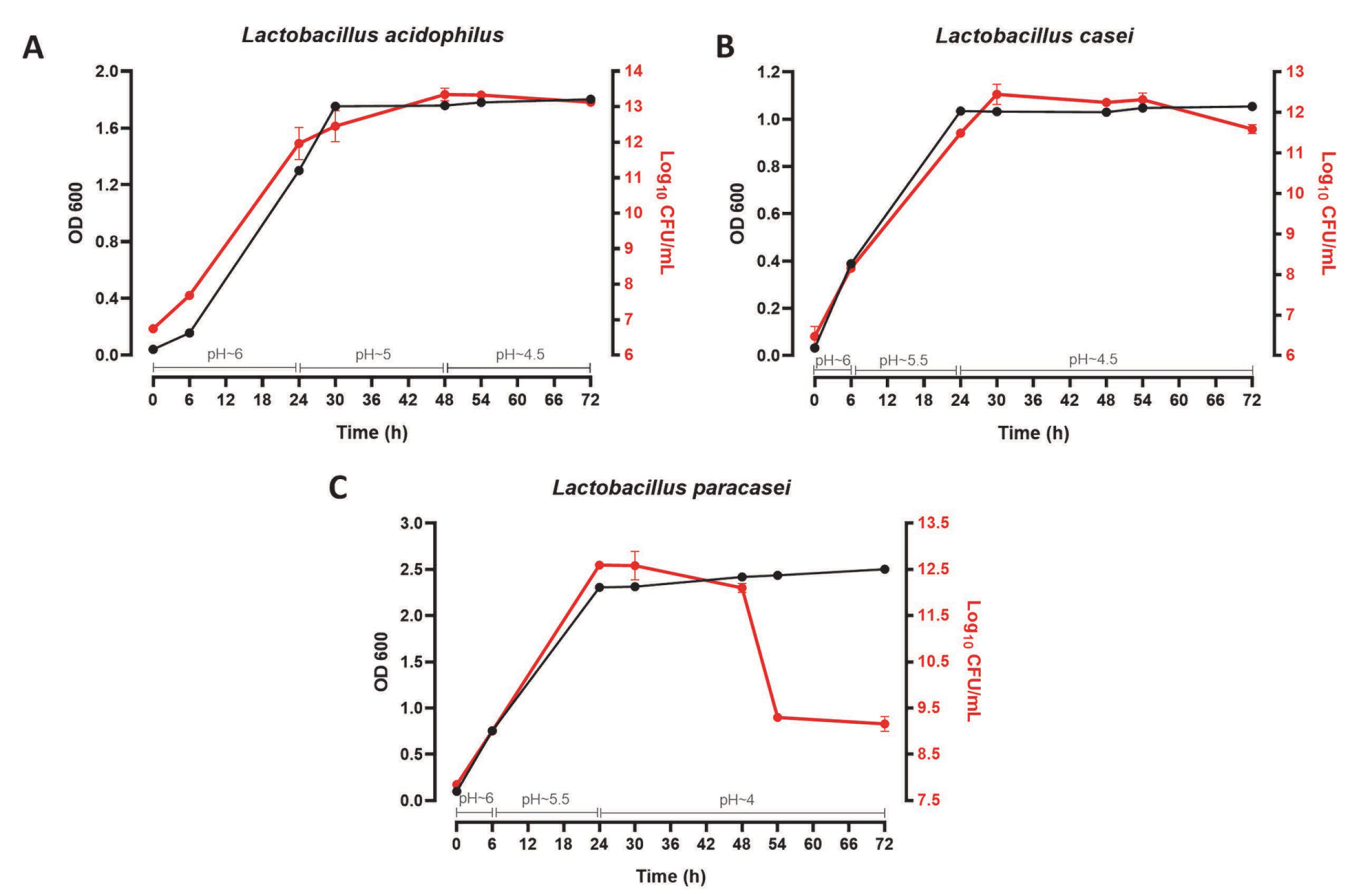
72-hour growth curve of *Lactobacillus acidophilus* (A); *L. casei* (B); and *L. paracasei* (C) probiotic bacteria. OD, optical density.

As observed in Figure 1, the growth curve of three *Lactobacillus* probiotic bacteria was drawn over 72 hours by two methods of optical absorption reading (OD 600) and colony count (Log_10_ CFU/mL). The pH of the culture medium was also determined in this time range. For the first 24 hours after cultivation, *L. acidophilus* (OD ~ 1.6 - Log_10_ CFU/mL ~ 13), *L. casei* (OD ~ 1 - Log_10_ CFU/mL ~ 12), and *L. paracasei* (OD ~ 2.5 - Log_10_ /mL ~ 12) bacteria were in the logarithmic growth phase, and the pH of their medium was about 6, 5.5 - 6, and 5.5 - 6, respectively. Within 24 to 48 hours, all three strains were in the stationary growth phase, and the pH of the medium was around 5, 4.5, and 4 for *L. acidophilus*, *L. casei*, and *L. paracasei*, respectively. In 72 hours, both *L. acidophilus* and *L. casei* entered the death phase (the pH of their culture medium: ~ 4.5). Nevertheless, *L. paracasei* entered this phase after 48 hours (the pH of the culture medium: ~ 4). Therefore, CFSs were isolated after 24 and 48 hours of cultivation.

### 4.2. Investigation and Comparison of the Antibacterial Effect of LCFSs

As observed in Table 1, the MIC of 24-hour acidic and neutral supernatants of *L. acidophilus* and *L. casei* on the *S. mutans* bacteria growth was 50% (v/v); however, *L. paracasei* showed a MIC of 12.5% (v/v) and 50% (v/v) with its acidic and neutral supernatants, respectively. In both acidic and neutral states, the aforementioned values for *A. actinomycetemcomitans* bacteria were 0% (no antibacterial activity) and 50% (v/v) for *L. acidophilus* and *L. casei*, respectively. Nonetheless, *L. paracasei* had a MIC of 25% (v/v) with acidic and 0% (no antibacterial activity) with neutral supernatants. The MBC was measured only in the 24-hour acidic supernatant of *L. paracasei* at a concentration of 50% (v/v). According to Table 1, the MIC of the 48-hour supernatant of *L. acidophilus* on the growth of *S. mutans* bacteria was 25% (v/v) (when prepared in both acidic and neutral conditions). Although the 48-hour supernatants of both *L. casei* and *L. paracasei* showed a MIC of 50% (v/v) with neutral LCFSs, they had MIC of 25% (v/v) and 12.5% (v/v) with acidic LCFSs, respectively. The aforementioned value for *A. actinomycetemcomitans* bacteria was 50% (v/v) for both acidic and neutral LCFSs derived from *L. acidophilus* and *L. casei*. Nevertheless, *L. paracasei* showed a MIC of 25% (v/v) with acidic and 0% (no antibacterial activity) with neutral LCFSs. Additionally, Table 1 shows that the MBC of both acidic and neutral 48-hour LCFSs of *L. acidophilus* and *L. casei* on *S. mutans* bacteria were 25% and 50% (v/v), respectively. This value for *L. paracasei* was 12.5% (v/v) with acidic and 0% (no antibacterial activity) with neutral supernatants. Furthermore, the MBC of 48-hour supernatants of *L. acidophilus*, *L. casei*, and *L. paracasei* on *A. actinomycetemcomitans* bacteria was equivalent to MIC.

**Table 1.** Antibacterial Effect of 24- and 48-Hour Acidic and Neutral Lyophilized Cell-Free Supernatants of Three Probiotic Bacteria, Including *Lactobacillus acidophilus*, *L. casei*, and *L. paracasei*, on Oral Caries Strain *Streptococcus mutans* and Oral Periodontal Strain *Aggregatibacter actinomycetemcomitans* ^a^

	24-Hour Lyophilized Cell-Free Supernatants						48-Hour Lyophilized Cell-Free Supernatants					
	*L. acidophilus*		*L. casei*		*L. paracasei*		*L. acidophilus*		*L. casei*		*L. paracasei*	
	pH = 4.3	pH = 7.2	pH = 4.3	pH = 7.2	pH = 3.6	pH = 7.2	pH = 4.3	pH = 7.2	pH = 4.3	pH = 7.2	pH = 3.6	pH = 7.2
***S. mutans*** **(% v/v)**												
MIC	50	50	50	50	12.5	50	25	25	25	50	12.5	50
MBC	-	-	-	-	50	-	25	25	50	50	12.5	-
***A. ** **actinomycetemcomitans*** ** (% v/v)**												
MIC	-	-	50	50	25	-	50	50	50	50	25	-
MBC	-	-	-	-	50	-	50	50	50	50	25	-

Abbreviations: MIC, minimum inhibitory concentration; MBC, minimum bactericidal concentration.

^a^ -: No antibacterial activity.

### 4.3. Investigation and Comparison of the Effect of LCFSs on the Viability of PDLSCs

**Figure 2.**
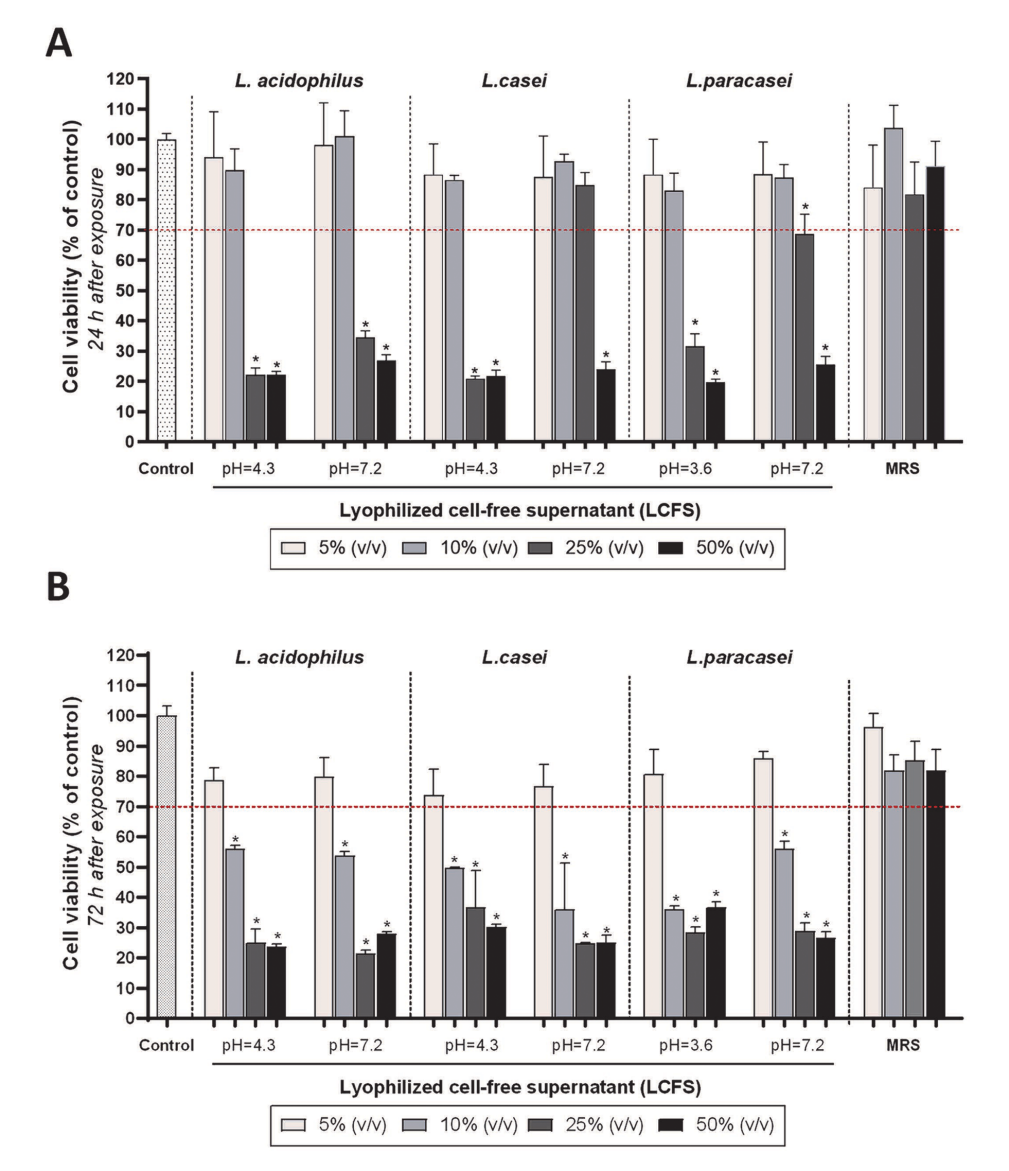
Quantitative investigation of the effect of different concentrations of lyophilized cell-free supernatants on the survival and proliferation of periodontal ligament stem cells, 24 (A); and 72 (B) hours after treatment. Stars on the columns indicate the statistical significance of the difference in the survival percentage of the target group compared to the control group (100% survival) (P < 0.05). The red dashed line represents the limit of a 30% decrease in survival, which according to the definition (the International Organization for Standardization-10993-5), indicates cytotoxicity.

To investigate and compare the acute cytotoxicity, the PDLSCs were exposed to the 48-hour acidic and neutral LCFSs of all three *Lactobacillus* probiotic bacteria for 24 hours. As observed in Figure 2A, 5% and 10% (v/v) acidic and neutral LCFSs derived from three probiotic bacteria had no cytotoxic effect on PDLSCs (P > 0.05). The effect of both concentrations on the cell viability was similar to each other and with the negative control group (no cytotoxicity, 100% survival) (P > 0.05). However, the concentrations of 25% and 50% (v/v) lyophilized supernatants caused a significant decrease in the percentage of cell survival to less than 70% (P < 0.05).

The PDLSCs were also exposed to the 48-hour acidic and neutral LCFSs of all three *Lactobacillus* probiotic bacteria for 72 hours to evaluate the chronic cytotoxicity. Although there was no significant toxic effect on PDLSCs when the cells were exposed to 5% (v/v) either acidic or neutral studied LCFSs (P > 0.05), 10%, 25%, and 50% (v/v) lyophilized supernatants significantly reduced cell viability (< 70% viability) (P < 0.05). It should be noted that no statistically significant difference was observed between all four concentrations of MRS culture media as a control group each other and with the negative control group (P > 0.05) (Figure 2B). According to the International Organization for Standardization-10993-5, the 5% (v/v) LCFSs are non-cytotoxic since they cause less than a 30% reduction in cell survival (15). Therefore, the subsequent assays in this study were conducted using the 5% (v/v) LCFSs.

### 4.4. Investigation and Comparison of the Effect of LCFSs on the Migration of PDLSCs

**Figure 3.**
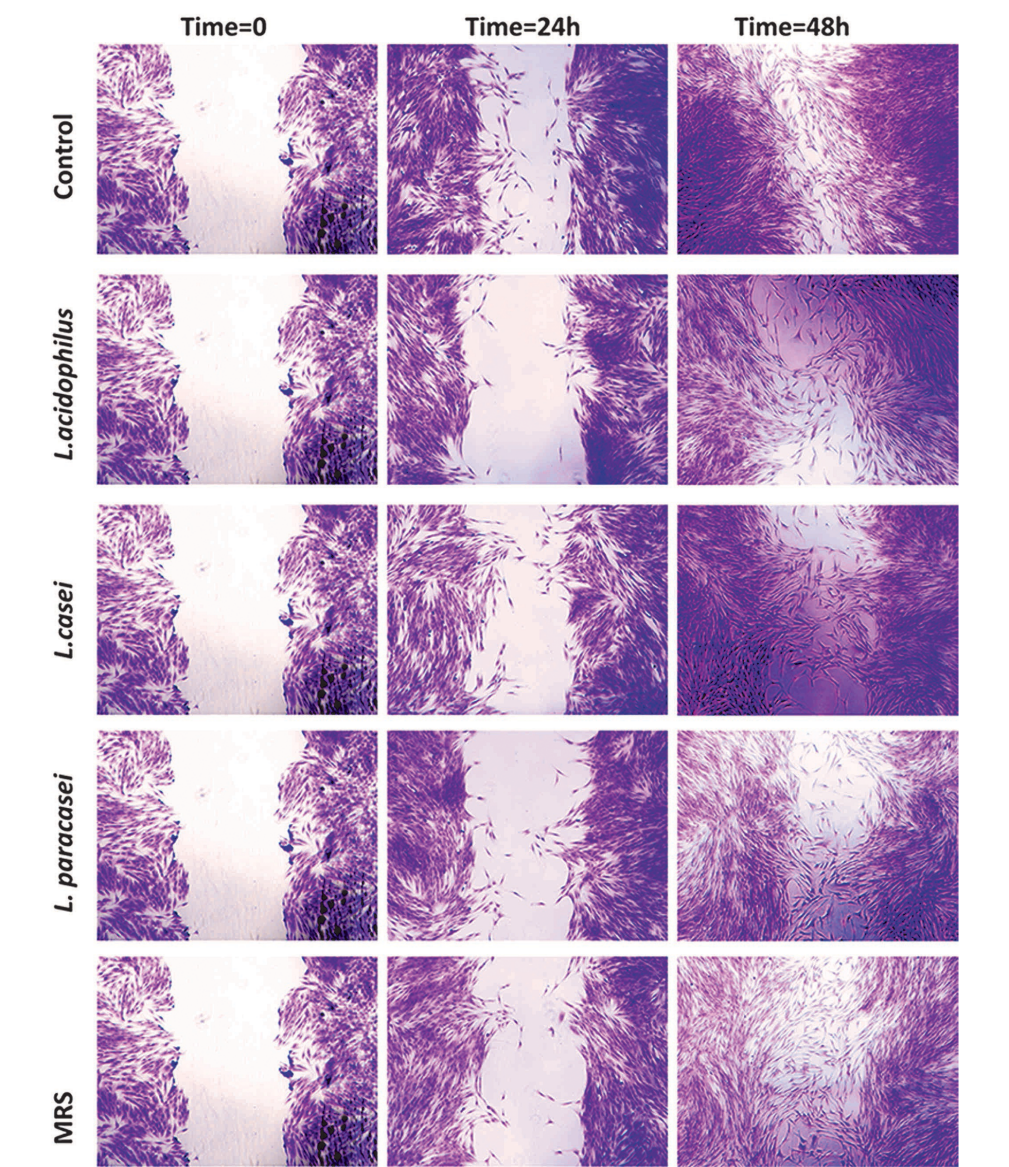
Qualitative investigation of the effect of lyophilized cell-free supernatants of three probiotic bacteria on the migration of proliferation of periodontal ligament stem cells at 0, 24, and 48 hours after treatment

The wound healing assay was performed to determine the impact of 5% (v/v) 48-hour neutral LCFSs of three studied probiotic bacteria on the migration capability of PDLSCs. As observed in Figure 3, after 24 or 48 hours of exposure, no statistically significant difference was observed between the effect of LCFSs with each other and with the negative control group on the migration capability of PDLSCs (P > 0.05).

### 4.5. Investigation and Comparison of the Antioxidant Effects of LCFSs

**Figure 4.**
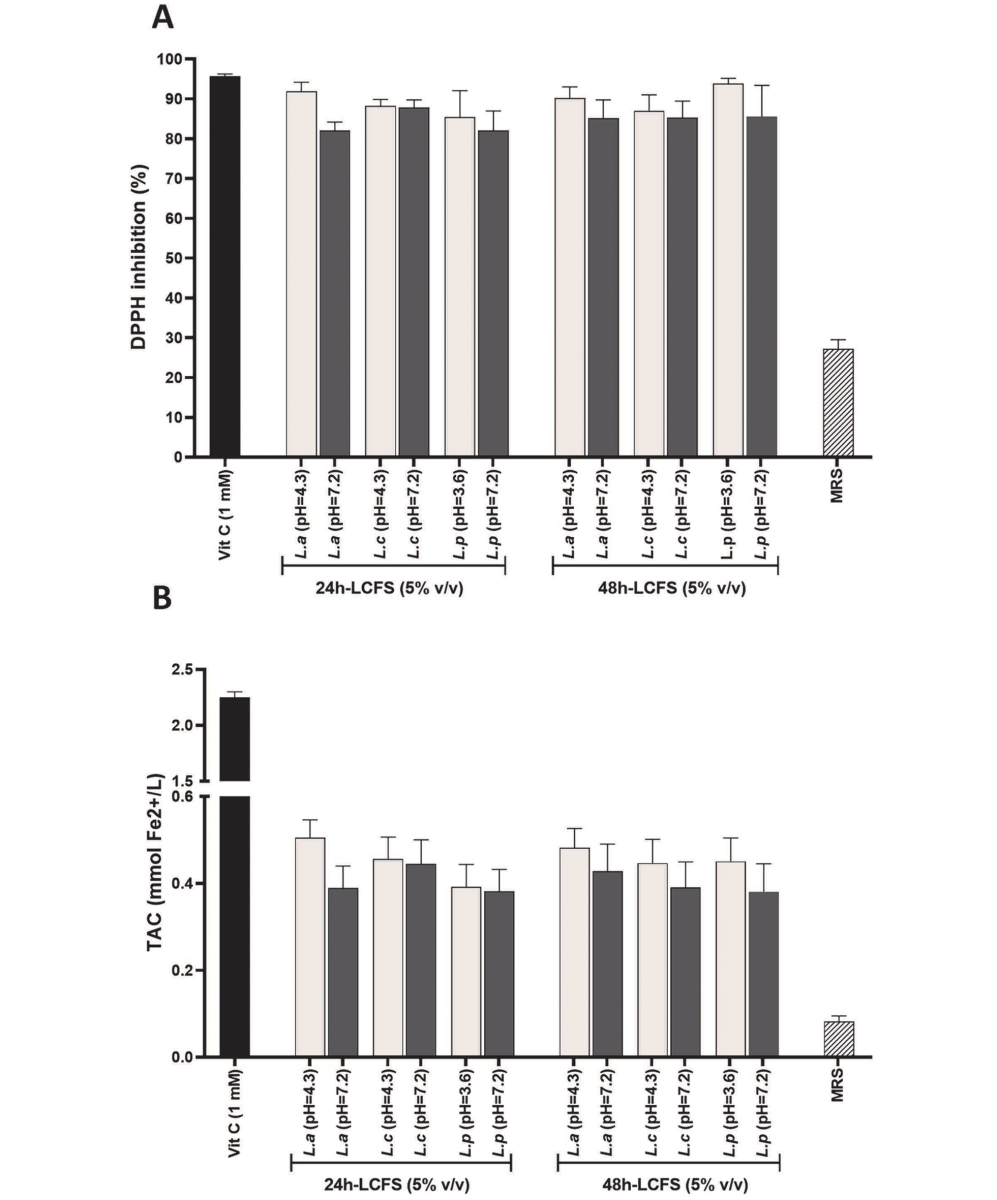
Quantitative investigation and comparison of antioxidant effects of 5% (v/v) of 24- and 48-hour acidic and neutral lyophilized cell-free supernatants by 2,2-diphenyl-1-picrylhydrazyl assay (A); and ferric reducing/antioxidant power assay (B). DPPH, 2,2-diphenyl-1-picrylhydrazyl; LCFSs, lyophilized cell-free supernatants; TAC, total antioxidant capacity. Vitamin C and De Man, Rogosa, and Sharpe are positive and negative controls, respectively.

As observed in Figure 4A, ascorbic acid (vitamin C) with a concentration of 1 mM as a positive control group inhibited DPPH free radicals by nearly 100%. Similarly, 24- and 48-hour acidic and neutral supernatants with a concentration of 5% (v/v) inhibited DPPH free radical (almost 100%) with no statistically significant difference between each other and the control group (P > 0.05). It should be noted that the lower antioxidant effect of MRS as a negative control group, compared to LCFSs and positive control, was highly significant (P < 0.05). According to Figure 4B, vitamin C with a concentration of 1 mM as a positive control group reduced about 2 mM of divalent iron. Furthermore, 24- and 48-hour acidic and neutral LCFSs with a concentration of 5% (v/v) reduced about 0.6 mM of iron without statistically significant difference with each other (P > 0.05). Similar to the DPPH test, LCFSs, and positive control showed a highly significant (P < 0.05) antioxidant effect compared to MRS.

## 5. Discussion

The role of different periodontopathogens, such as *A. actinomycetemcomitans*, in chronic and progressive periodontitis has been proven in previous studies (16, 17). Due to the protective role of probiotics (18, 19), the current study aimed to mark a novel investigation by comparatively examining both the antioxidant activity and the antimicrobial effect of mentioned LCFSs (i.e., *L. acidophilus*, *L. casei*, and *L. paracasei*) against the oral bacteria (i.e., *A. actinomycetemcomitans* and *S. mutans*) and the effects on the cell proliferation and migration of PDLSCs.

As one of the defining characteristics of probiotics is their antibacterial properties, demonstrating the antibacterial capabilities of lactobacilli under investigation serves as the validation of their probiotic nature (20). After preparing and lyophilizing the supernatants (at both acidic and neutral pH), their antibacterial effects were investigated on oral caries strain *S. mutans* and oral periodontal strain *A. actinomycetemcomitans*. The obtained results showed that the supernatants of *L. acidophilus* extracted after 48 hours had the strongest antibacterial properties among the lactobacilli studied.

In addition, consistent with the results of Taşkın and Akköprü’s study, in the present study, 48-hour supernatants, independent of their pH, showed further antibacterial properties than 24-hour supernatants (21). This difference could be due to the accumulation of antibacterial compounds over time. Consistently, Koll-Klais et al.’s research results confirm the antibacterial properties of lactobacilli against periodontopathogens, such as *A. actinomycetemcomitans*, *S. mutans*, *Porphyromonas gingivalis*, and *Prevotella intermedia*. This study used 10 different strains of lactobacilli, such as *L. paracasei*, *Lactobacillus gasri*, and *Lactobacillus rhamnosus*. These lactobacilli showed different inhibitory effects on periodontopathogens. For instance, 96%, 88%, 82%, and 65% of them possess inhibitory effects on *S. mutans*, *A. actinomycetemcomitans*, *P. gingivalis*, and *P. intermedia*, respectively (22).

Moreover, Rossoni et al. showed that most lactobacilli strains isolated from caries-free oral cavities could release bioactive substances that inhibit the growth of *S. mutans*. In this study, some strains, such as *L. paracasei* and *Lactobacillus fermentum*, were mentioned as lactobacilli that belong to the natural flora of the mouth (23). The antimicrobial effect of CFSs can be due to the presence of acetic acid and lactic acid. These acids show antibacterial properties in their protonated form at low acidity, preferably pH < pKa. Under these conditions, proteins and nucleic acids are affected by the protonated acid as soon as they enter the cell. After breaking down the acid, the cell spends energy to restore the acidity of the cytoplasm. Other metabolites and complex products, such as diacetyl and hydrogen peroxide, also have antimicrobial properties (24). Hence, it can be inferred that the use of CFSs offers a greater benefit compared to employing purified compounds due to the fact that supernatants consist of a combination of diverse metabolites, leading to a wide range of antimicrobial effects.

Given the demonstration of the acceptable antibacterial properties of LCFSs, it is crucial to confirm their non-toxicity on human cells, particularly PDLSCs. In the current study, according to the MTT results, the acute and chronic cytotoxicity of 48-hour acidic and neutral supernatants with a concentration of 5% (v/v) was not observed on human PDLSCs. This finding is consistent with Maqsood et al.’s study findings in which they investigated the cytotoxicity of the LCFSs of *Lactobacillus rhamnosus* and *L. acidophilus* on a human monocytic cell line (THP-1 differentiated with PMA). They reported that concentrations higher than 10% (v/v) of LCFSs showed cytotoxicity (25).

Furthermore, according to the present study’s results, 5% (v/v) LCFSs of all three strains did not show any negative effects on the cellular migration of human PDLSCs. It is noteworthy to mention that utilizing the cell lysate instead of the supernatants might deliver different results. For instance, Han et al. studied the effect of *Lactobacillus reuteri* extracts on the migration of mice gingival mesenchymal stem cells. According to the aforementioned study’s results, 50 μg/mL *Lactobacillus* cell lysate promoted the process of wound healing via the PI3K (phosphatidylinositide 3-kinase)/ AKT (protein kinase B), β-catenin/ TGFβ1 (transforming growth factor beta 1) pathway (26).

In the present study, the antioxidant activity of LCFSs was also investigated using two antioxidant assays, namely DPPH and FRAP. The test was carried out utilizing the 5% (v/v) 24- and 48-hour acidic and neutral supernatants. The results showed that the studied LCFSs, without statistically significant differences with each other, inhibited DPPH free radicals and reduced divalent iron. Their antioxidant capacity was measured, especially high in the DPPH test and similar to 1 mM vitamin C.

In agreement with the current study’s results, Sornsenee et al. in 2021 reported that the supernatants of *L. acidophilus*, *L. casei*, *Lactococcus lactis*, and *Lactobacillus reuteri* possessed antioxidant activity and attributed the observed activity to phenolic and flavonoid compounds (27). Moreover, in addition to confirming the antioxidant properties of probiotics, Wang et al. proposed several possible mechanisms of action for how antioxidants might work. According to the aforementioned study’s results, probiotics might modulate the oxidation state of the host through the ability to chelate metal ions and antioxidant systems. Since metal ions, such as Fe^2+^ and Cu^2+^, might increase free radicals by catalyzing oxidation in the body, their chelation can reduce the production of free radicals. Furthermore, probiotics might regulate the signaling pathways of reactive oxygen species-producing enzymes and gut microbiota (28).

### 5.1. Conclusions

Conclusively, the present study’s results demonstrated that 5% (v/v) 48-hour acidic and neutral supernatants of three probiotic strains possess a significant antibacterial effect on two pathogenic oral bacteria with an essential role in periodontitis progression; however, they exert no cytotoxic effects on PDLSCs with an essential role in periodontitis prevention. Their antioxidant capacity at this concentration was also measured, especially high in the DPPH test and similar to vitamin C.

## Data Availability

The dataset presented in the study is available on request from the corresponding author during submission or after publication.
